# NLRP3 inflammasome activity is upregulated in an *in-vitro* model of COPD exacerbation

**DOI:** 10.1371/journal.pone.0214622

**Published:** 2019-05-21

**Authors:** Noy Nachmias, Sheila Langier, Rafael Y. Brzezinski, Matan Siterman, Moshe Stark, Sara Etkin, Avital Avriel, Yehuda Schwarz, Shani Shenhar-Tsarfaty, Amir Bar-Shai

**Affiliations:** 1 The Division of Pulmonary Medicine, Barzilai Medical Center, Faculty of Health Sciences, Ben-Gurion University, Ashkelon, Israel; 2 Department of Internal Medicine "C, "D and "E, The Tel Aviv Sourasky Medical Center, Tel Aviv, affiliated to the Sackler Faculty of Medicine, Tel Aviv University, Tel Aviv, Israel; 3 The Pulmonary Institute, Tel-Aviv Sourasky Medical Center and Sackler Faculty of Medicine, Tel Aviv University, Tel Aviv, Israel; 4 Neufeld Cardiac Research Institute, Sackler Faculty of Medicine, Tel Aviv University, Israel; Tamman Cardiovascular Research Institute, Leviev Heart Center, Sheba Medical Center, Tel-Hashomer, Israel; University of the Pacific, UNITED STATES

## Abstract

**Background:**

Chronic obstructive pulmonary disease (COPD) is an inflammatory disease characterized by a progressive and irreversible deterioration of lung function. Exacerbations of COPD have prolonged negative effects on pulmonary function and a major impact on health status and outcomes. NLRP3 inflammasome is a cardinal component of the inflammatory response, with marked evidence in stable and exacerbations of COPD. The aim of our study was to evaluate the NLRP3 inflammasome activity during COPD exacerbation by using an in vitro model.

**Methods:**

A549 cells were stimulated with different concentrations (10%, 4%, 2%) of cigarette smoke extract (CSE) with or without LPS (0.1μg/ml) for 24 hours. Cell viability was assessed by using XTT test. Levels of inflammatory cytokines (IL-8, MCP-1, and IL-1β) were measured by ELISA and the activity level of NLRP-3 was evaluated by flow cytometry.

**Results:**

Cells exposed to CSE present an increase in inflammatory cytokines (IL-8 and MCP-1) production in a dose-dependent manner. Incubation with LPS to these cells results in higher levels of IL-8 and MCP-1 compared to stimulation of CSE alone. NLRP3 inflammasome activity and IL-1β levels were significantly increased in cells exposed to both CSE and LPS compared to CSE alone.

**Conclusions:**

NLRP3 inflammasome is upregulated in an in-vitro model of COPD and COPD exacerbation. Our findings provide novel biomarkers for COPD exacerbation and may present new targets for future research.

## Introduction

Chronic obstructive pulmonary disease (COPD) is a common respiratory condition associated with cigarette smoke exposure and characterized by airflow limitation that is usually progressive. COPD is also associated with an enhanced chronic inflammatory response in the airways, lungs, and serum [1,2]. The clinical pattern of COPD is characterized by episodes of symptom worsening termed exacerbations. COPD exacerbations have a major role in deterioration in health status and disease progression, as well as a decline in lung function after each exacerbation [3]. Furthermore, COPD exacerbations are the most prevalent cause of mortality and cardiovascular events in COPD patients, and the risk of mortality is increasing with each hospitalization due to an exacerbation episode [3].

The inflammatory process and its regulation during COPD exacerbation are remarkably complex. The leading cause to exacerbation is the immune response to bacterial pathogens such as Staphylococcus aureus, Streptococcus pneumoniae, Haemophilus influenzae, Klebsiella pneumoniae, Chlamydia pneumoniae, Mycobacterium tuberculosis and viral pathogens, such as influenza A and respiratory syncytial virus [4, 5]. However, since there is a clinical heterogeneity in COPD exacerbations, researchers try to find biomarkers that would help clinicians to tailor the most appropriate treatment. Many biomarkers were investigated including C- reactive protein (CRP), sputum or blood eosinophils, IL-1 β, caspase-1, IL-18, CXCL10, IL-8\CXCL8 and MCP-1 [5–10].

Recently many studies investigated the role of NLR (Nod-like receptors) family in COPD and the NLRP-3 (NLR containing a Pyrin domain 3) [10–16]. Briefly, the activation of NLRP-3 inflammasome complex leads to the activation of Caspase-1 which causes the cleavage and maturation of the pro-inflammatory cytokines IL-1β and IL-18 [15]. Likewise, the signaling pathway of NLRP-3 is found to be a major mediator of immune response to exposure of cigarette smoke and airborne insults, through activation of toll-like receptors on epithelial cells and activation of immunological cells such as neutrophils and dendritic cells [16, 17].

While there is abundant information regarding the NLRP-3 role in stable COPD by using animal models [11, 12, 13] and COPD patients`sputum [8,12–14], there is limited information about NLRP-3 during COPD exacerbation [6,8,12].

Here, we aim to investigate the role of NLRP3 in an in-vitro model which was designed to mimic COPD exacerbation.

## Methods

### Cell culture

A549 cells, pulmonary adenocarcinoma derived cell line [18], purchased directly from Sigma- Aldrich (catalog number 86012804) and cultured in Dulbecco’s modified eagle’s medium (DMEM), (Biological Industries, Beit Haemek, Israel) with 10% fetal calf serum (FCS), 100 units/ml penicillin, and 100 mg/ml streptomycin, under a humidified atmosphere (5% CO2 plus 95% air) at 37°C. Treatments included 2%, 4% and 10% CSE. LPS was added to the cultures to stimulate COPD exacerbation condition [6].

### Preparation of aqueous cigarette smoke extract (10%)

One research cigarette (3R4F, Kentucky tobacco research center) was bubble into 10 ml of culture media supplemented with 1% FBS through a smoking apparatus at a rate of one cigarette/minute. CSE was sterile filtered through a 0.22 μm filter. CSE preparation was standardized by measuring the absorbance (OD 0.74 ± 0.05) at a wavelength of 320 nm [19, 20].

### Evaluation of inflammatory response of IL-8, MCP-1 and IL-1β levels

To evaluate CSE and LPS cytotoxicity during COPD exacerbation model, we measured levels of IL-8 and MCP-1. These cytokines were found to be increased in BAL (Broncho-Alveolar Lavage) of COPD patients [5, 7, 8]. Briefly, Enzyme-Linked immunosorbent assay (ELISA) was performed in the supernatant of the cell cultures for human IL-8, MCP-1 and IL-1β, according to the manufacturer (e-bioscience coating ELISA kit).

### Evaluation of NLRP3 activity

NLRP3 protein expression was measured by flow cytometry, according to the instructions of the manufacturer. Briefly, A-549 cell suspension adjusted to a concentration of 1–5 x 106 cells/mL in ice-cold PBS, 10% FCS 1% sodium azide. Cells were stained in an Anti-Human NLRP3 conjugated monoclonal antibody. To facilitate intracellular staining, cells were fixed with Flow Cytometry Fixation Buffer and permeabilized with Flow Cytometry wash Buffer. Then, cells centrifuged sufficiently, and the supernatant fluid was removed with minimum loss of cells.

### Statistical analysis

The results are presented as means ± SEM. Analysis of variance (ANOVA) was conducted with the Bonferroni post hoc comparison tests and used to compare the levels of inflammatory biomarkers and to evaluate the differences between each group. P value < 5% was considered statistically significant.

## Results

### Exposure to cigarrete smoke cause a depletion in cell viability

**Fig 1:**
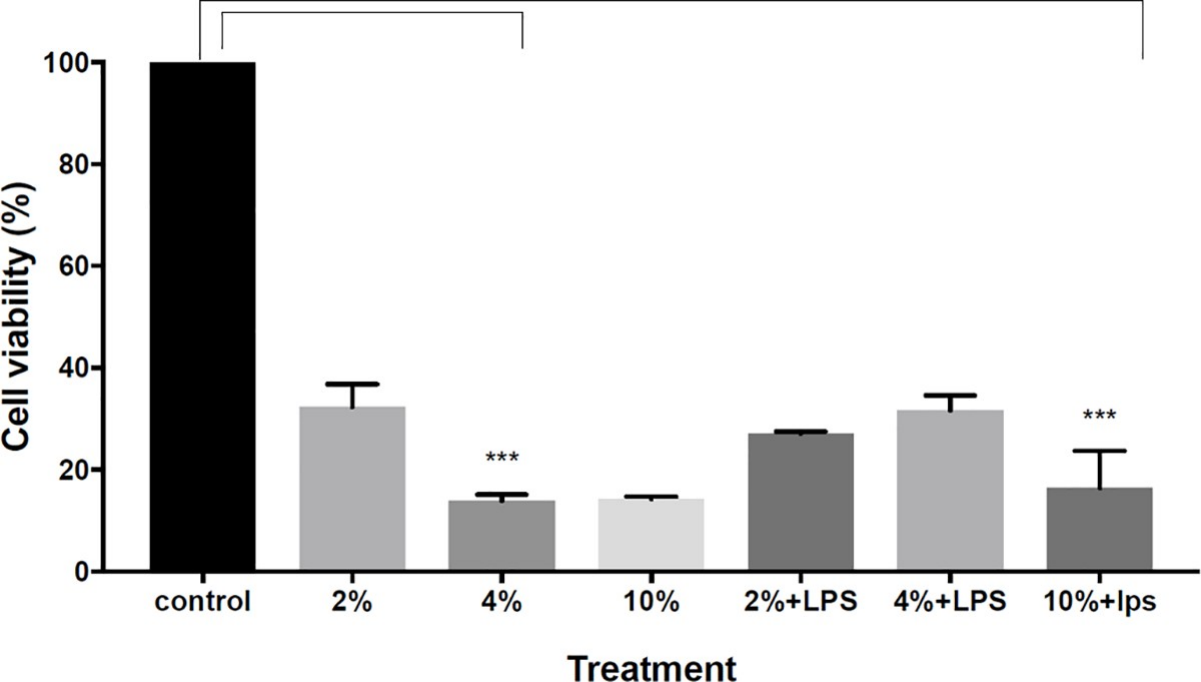
The effect of cigarette smoke exposure with or without LPS exposure on cell`s viability: A-549 cells were incubated for 24 hours with CSE of 2%, 4% and 10% or with the combination of LPS (0.1 μg\ml). Control group incubated with medium alone (FCS 1%). Cell viability evaluated by using XTT test. The data represent the mean ± SEM of 3 experiments***P value<0.0001.

According to Fig 1, exposure of A-549 cells to CSE showed reduced cell viability compared to the non-exposed cells (control group). The magnitude of cell depletion was partially correlated with the concentration of cigarette smoke extract (Fig 1).

In order to evaluate the inflammatory response during COPD exacerbation, we added LPS to the cell cultures, as an in vitro model of the disease. While the addition of LPS caused a significant cell depletion in all CSE concentration (p value<0.001) it was not in a dose-dependent manner, with no significant difference between CSE of 2% and 4% (p value = 0.0649). The most profound effect was in cells exposed to 10% CSE with the addition of LPS (Fig 1).

### The inflammatory response in COPD and COPD exacerbation model

**Fig 2:**
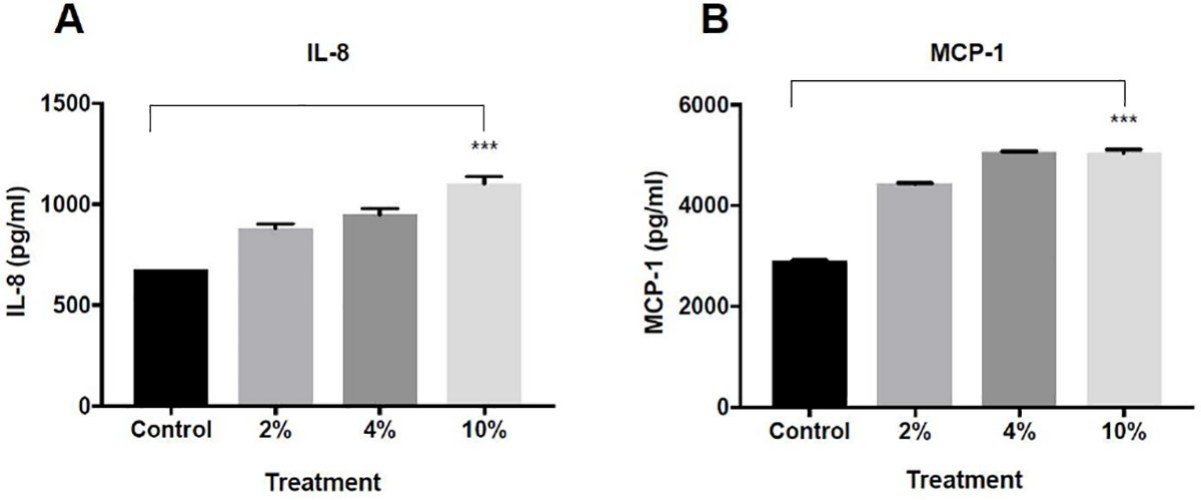
The effect of CSE in different concentrations on the inflammatory response in a COPD in-vitro model: A-549 cells were incubated for 24 hours with CSE of 2%, 4% and 10% or with medium alone (FCS 1%, control group). The culture supernatants were collected and the concentrations of IL-8 (A) and MCP-1(B) were measured by ELISA. The data represent the mean ± SEM of 3 experiments. ***P value<0.0001.

The levels of IL-8 and MCP-1 were measured to evaluate the inflammatory effect in COPD. In cells exposed to CSE solely, the concentration of IL-8 and MCP-1 increased significantly, (P <0.001) (Fig 2A and 2B), though the differences between 2% and 4% concentration in the production of IL-8 (Fig 2A) and between 4% and 10% in the MCP-1 group (Fig 2B) were not significant.

**Fig 3:**
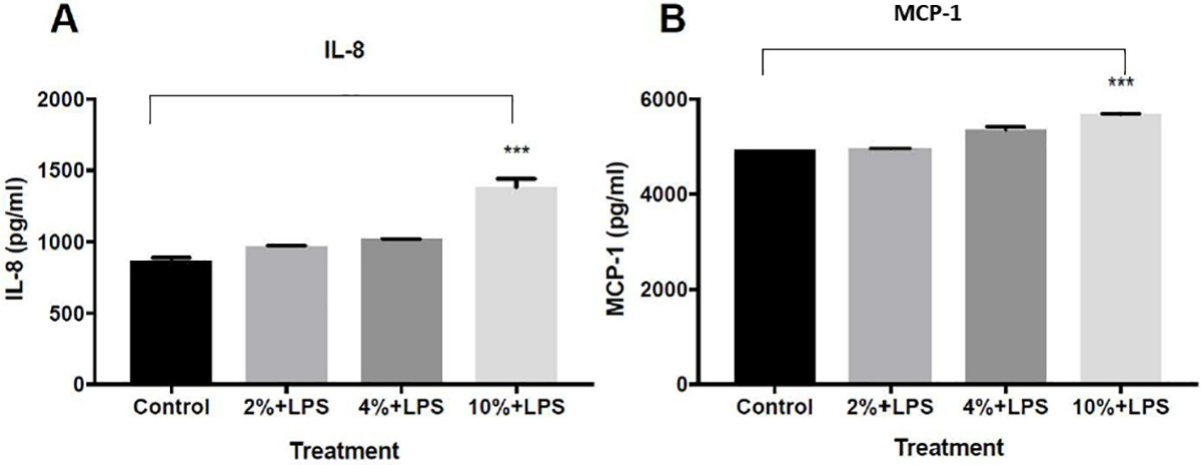
The effect of CSE in different concentrations on the inflammatory response in a COPD Exacerbation in vitro model: A-549 cells were incubated for 24 hours with LPS (0.1μg\ml) and CSE of 2%, 4% and 10% or with LPS alone (control group). The culture supernatants were collected and the concentrations of IL-8 and MCP-1 were measured by ELISA. The data represent the mean of 3 experiments ± SEM.***P value < 0.0001.

The addition of LPS exposure (Fig 3) induced an increase in IL-8 and MCP-1 (Fig 3A and 3B). The increment in levels of both cytokines was significant (P value<0.001 for both). Post hoc analysis reveals that the most profound effect was observed in CSE 10% and LPS, while there was no significant difference between control group to 2% CSE with LPS treatment.

### Inflammasome activity in COPD and COPD exacerbation in vitro model

**Fig 4:**
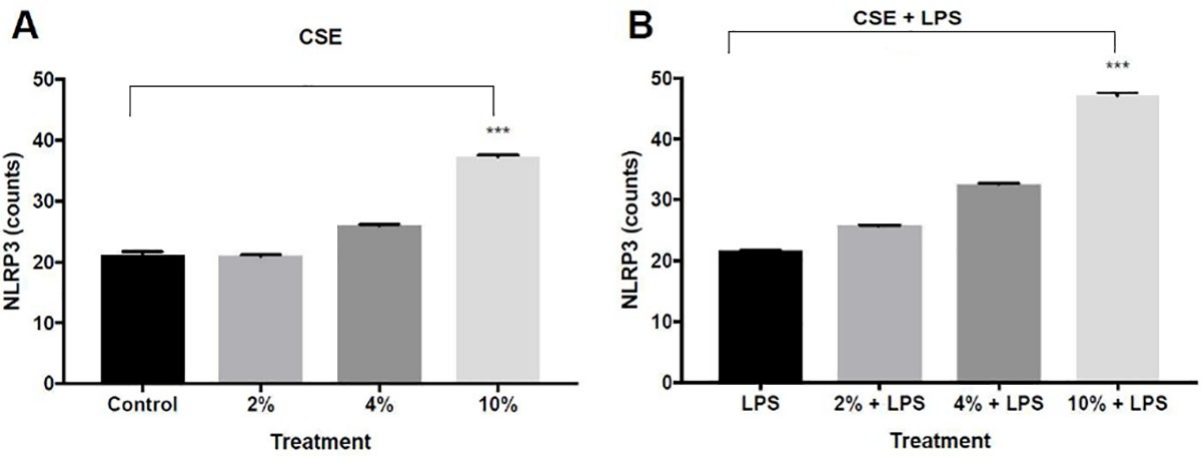
**The effect of CSE with different concentrations on the activity of inflammasome as a part of COPD and COPD exacerbation in vitro model**: A-549 cells were incubated for 24 hours with the following treatments: (A)- CSE of 2%, 4% and 10% or with medium alone (control group), (B)- CSE of 2%, 4% and 10% and LPS (0.1μg\ml) or with LPS (0.1μg\ml) alone (control group), then culture supernatants were collected. NLRP3 level was evaluated by Flow cytometry. The data represent the mean of 3 experiments ± SEM. *** P value < 0.0001.

Levels of intra-cellular NLRP3 protein, were measured in cells exposed to CSE. No change was observed in NLRP3 expression between the control group and CSE 2% (Fig 4A). However, a significant increase in the inflammasome response was found in cells exposed to CSE in concentrations of 4% or higher (Fig 4A). The addition of LPS increase NLRP3 activation in a dose-dependent manner (Fig 4B), the mean NLRP3 counts were 21.63 (+ 0.18), 25.69 (±0.21), 32.54 (±0.28) 47.07 (±0.69) for control, 2%,4%.10% CSE, respectively.

**Fig 5:**
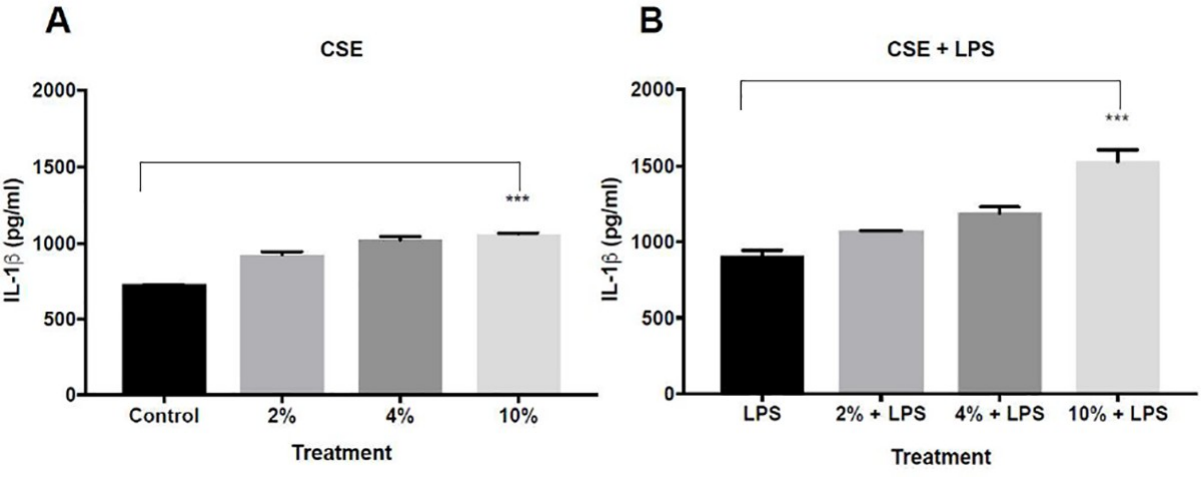
**The effect of CSE with different concentrations on the activity of IL-1β as a part of COPD and COPD exacerbation in vitro model**: A-549 cells were incubated for 24 hours with the following treatments:(A)- CSE of 2%, 4% and 10% or with medium alone (FCS 1%, control group), (B)- LPS (0.1μg\ml) and CSE of 2%, 4% and 10% or with LPS alone (control group). The culture supernatants were collected. The concentration of IL-1β was measured by ELISA. The data represent the mean of 3 experiments ± SEM. ***P value < 0.0001.

Since IL-1β production depends on NLRP-3 expression [10, 11], we measured its levels in the supernatant of the cell cultures. High levels of IL-1β were measured in cells exposed to CSE 4% and 10% versus control (A-549 cells without exposure to CSE) (Fig 5A).

Concordant to NLRP3 activity, the concentration of IL-1β also increased following exposure to CSE+ LPS (Fig 5A and 5B).

## Discussion

Our study offers new in vitro model for COPD and COPD exacerbation, our model demonstrates two major findings: first, NLRP3 and its functional products are up-regulated in an *in vitro* model of COPD. Second, this up-regulation is augmented in COPD exacerbation.

Numerous in-vitro and animal models for stable COPD are in use [10,11,17, 20] compared to the limited data for COPD exacerbation models [6,21]. In our in vitro model, we exposed A-459 cells to cigarette smoke in different concentrations, as previously established as an in vitro model for COPD [6, 19, 20]. A-549 cell line is derived from a human pulmonary adenocarcinoma with biochemical and morphological features of an alveolar cell and is frequently used in experimental models [6, 20, 21]. The addition of LPS, designed to mimic COPD exacerbation as a part of our in vitro model. This addition is based on the fact that bacterial pathogens are the leading triggers to COPD exacerbation. and on other published studies [6, 22].

Our study supports the principal pathophysiology of cigarette smoke-induced inflammation in COPD which damage lung tissue by depletion of cell viability [4,6]. Although there wasn`t a significant difference between exposure to CSE 4% and CSE 10%, the overall effect on viability was found to be dose dependent, as CSE concentration increases. This observation may imply that the effect of CSE on cell viability reaches its maximal effect in the concentration of 4%.

Since MCP-1 and IL-8 are important mediators in the inflammatory response in COPD [4,6], our results strengthen the in-vitro model in this experiment for COPD and are in line with previous studies demonstrating an increase in these cytokines in stable COPD [4,5,13,17]. We found high levels of MCP-1 and IL-8 after exposure to CSE, and IL8 levels were also in correlation to CSE concentration. Concordant to cell viability experiment, this correlation was not found in MCP-1 concentration: the highest concentration of MCP-1 was in cells exposed to 4% of CSE and did not show further increase when exposing the cells to CSE 10%. We predict this is due to the maximal production level.

The inflammasome pathway in respiratory diseases and particularly in COPD was increasingly investigated in the last years, with a focus on stable COPD. The activation of NLRP-3 was found to be a key modulator of respiratory infections and airway inflammation and the assembly of NLRP-3 components`triggers a pro-inflammatory cell death mode [5]. Moreover, Pauwels et al. showed that IL-1β is an important component in cigarette smoke- induced inflammation and in COPD [10]. Similar to these results we found that exposing cell lines to CSE, representing stable COPD, results in activation of the inflammasome pathway. In our model, in addition to the increase in cell death and inflammatory cytokines, a higher NLRP-3 activity was observed in cells exposed to CSE. Furthermore, levels of IL-1β increased in a similar manner, suggesting that the production of IL-1β is a direct result of the NLRP-3 activation.

Given that exacerbation in COPD is an episode of an enhancement of the immune response, which causes an amplification of cell death [4,23], we found a greater decline in viability of cells after LPS exposure, an augmentation in the production of IL-8 and MCP-1, as well as a significant increase in NLRP3 activity and its product IL-1β.

Faner et al. found a high activity of NLRP3 and IL-1β in sputum samples taken from patients with infectious COPD exacerbation [12]. Two human studies looked at biomarkers during COPD exacerbation. Bafadel et al. investigated the expression of inflammatory biomarkers in patients`sputum during COPD exacerbation and found that IL-1β levels correlate significantly with COPD exacerbation due to bacterial infection [8], and recently, in a small cohort of COPD patients, levels of serum IL-1β and IL-17 were found elevated in COPD exacerbation compared to stable COPD and healthy controls [24]. Altogether, these studies validate the important role of NLRP3 during COPD exacerbation and are concordant with the results shown by our in -vitro model of COPD exacerbation.

Our study has some limitations which originate, primarily since it is based on an *in vitro* model. As such it cannot represent the multi-aspects of COPD exacerbation in real life. Other medical illnesses in COPD patients, response to therapies, different etiologies for exacerbation which may influence the results in the clinical settings. In addition, our model included only epithelial cells, without their factual environment, thus cannot evaluate downstream products, such as activation of innate immune cells which can further enhanced the upregulation of NLRP3.These effects should be addressed in future studies.

In conclusion, our study presents a possible role of epithelial cells' inflammasome in an in vitro model of COPD and COPD exacerbation. Further investigation in in-vivo models and in COPD patients is needed to support our findings in the clinical setting. Nevertheless, our results may lead to the foundation of NLRP-3 system as a novel biomarker in the diagnosis of COPD exacerbation and as a new target for therapies.

## Supporting information

S1 Table **The effect of cigarette smoke exposure with or without LPS exposure on cell`s viability:** A-549 cells were incubated for 24 hours with CSE of 2%, 4% and 10% or with the combination of LPS (0.1 μg\ml). Control group incubated with medium alone (FCS 1%). Cell viability evaluated by using XTT test. The data represent the mean ± SEM of 3 experiments
(XLSX)

S2 Table **The level of IL-8 and MCP-1 in response to cigarrete smoke exposure as an in vitro model of COPD:** A-549 cells were incubated for 24 hours with CSE of 2%, 4% and 10% or with medium alone (FCS 1%, control group). The culture supernatants were collected and the concentrations of IL-8 (A) and MCP-1(B) were measured by ELISA. The data represent the mean of 3 experiments ± SEM.
(XLSX)

S3 Table **The level of IL-8 and MCP-1 in response to cigarrete smoke exposure and LPS as an in vitro model of COPD exacerbation:** A-549 cells were incubated for 24 hours with LPS (0.1μg\ml) and CSE of 2%, 4% and 10% or with LPS alone (control group). The culture supernatants were collected and the concentrations of IL-8 and MCP-1 were measured by ELISA. The data represent the mean of 3 experiments ± SEM.
(XLSX)

S4 Table **The effect of CSE with different concentrations on the activity of IL-1β as a part of COPD and COPD exacerbation in vitro model**: A-549 cells were incubated for 24 hours with the following treatments:(A)- CSE of 2%, 4% and 10% or with medium alone (FCS 1%, control group), (B)- LPS (0.1μg\ml) and CSE of 2%, 4% and 10% or with LPS alone (control group). The culture supernatants were collected. The concentration of IL-1β was measured by ELISA. The data represent the mean of 3 experiments ± SEM.
(XLSX)

S5 Table **The level of NLRP3 inflammasome in different concentrations of CSE with or without exposure to LPS, as a part of an in vitro model for COPD and COPD exacerbation:** A-549 cells were incubated for 24 hours with the following treatments: (A)- CSE of 2%, 4% and 10% or with medium alone (control group), (B)- CSE of 2%, 4% and 10% and LPS (0.1μg\ml) or with LPS (0.1μg\ml) alone (control group), then culture supernatants were collected. NLRP3 level was evaluated by Flow cytometry. The data represent the mean of 3 experiments ± SEM.
(XLSX)
